# Adoption and Impact of the Maize Hybrid on the Livelihood of the Maize Growers: Some Policy Insights from Pakistan

**DOI:** 10.1155/2020/5959868

**Published:** 2020-01-31

**Authors:** Akhter Ali, AbduRahman Beshir Issa, Dil Bahadur Rahut

**Affiliations:** ^1^International Maize and Wheat Improvement Center (CIMMYT), CSI Complex, NARC, Park Road, Islamabad, Pakistan; ^2^International Maize and Wheat Improvement Center (CIMMYT), Kathmandu, Nepal; ^3^Socioeconomic Program, International Maize and Wheat Improvement Center (CIMMYT), Mexico City, Mexico

## Abstract

Despite hybrids being grown on 30–40 percent of the maize area in Pakistan, the retail price of hybrid maize seed is high in Pakistan compared with its neighbors in South Asia and beyond. Hence, this paper analyzes the adoption and impact of hybrid maize on livelihoods using a cross-sectional dataset collected from 822 maize growers in Pakistan. The data were collected from two types of farmers: adopters and nonadopters of hybrid maize, from four major provinces of Pakistan (Punjab, Sindh, KPK, and Balochistan). We use the bivariate probit to analyze the factors influencing the adoption of hybrid maize and the propensity score-matching (PSM) approach to analyze the impact of hybrid maize adoption on livelihood of maize growers, as PSM helps correct sample selection biasedness. The empirical result shows that farm size, farm and household assets, the level of education of farmers, access to market, and social networks positively influence the adoption of hybrid maize in Pakistan. The results from PSM revealed that hybrid maize adopters had higher grain yields in the range of 94–124 kgs per hectare as compared with nonadopters. Similarly household income levels were more in the range of Pakistani rupees 2,176–3,518, while the poverty levels were lower in the range of 2-3 percent for hybrid maize adopters. As hybrid maize adoption has had a positive impact on the livelihood of farmers, policies should aim to scale up the adoption of hybrid maize through enhancing the supply and lowering the seed cost through research and subsidy programs, thereby enabling poor farmers in remote areas to adopt hybrid maize varieties.

## 1. Introduction

In Pakistan, agriculture is the second most important sector of the economy (following textile and manufaturing industries) [1], and maize is the third most important cereal after wheat and rice. Approximately 30 percent of the land growing maize is used for hybrids, while 70 percent grows open-pollinated varieties (OPVs) (this indicates a huge expanding scope for maize hybrids in Pakistan). In Pakistan, 60 percent of aggregate maize production is used in the poultry feed sector, 25 percent in wet milling and other businesses, with the remainder utilized as nourishment for humans and animals [2]. The demand for maize is increasing because of these multiusages, which is stimulating farmers to invest further in maize production (in Pakistan, the price of maize hybrid seed is among the highest in South Asia, at 7-8 US$ per kilogram). As maize is a quick growing crop with the potential to produce a high quantity of grains per unit area [3], it could contribute to improving the livelihood of poor rural farmers, thereby moving them out of poverty.

Maize is mainly produced in the provinces of KPK and Punjab, although in recent years maize production has increased in traditionally non maize growing provinces like Sindh and at limited scale in Balochistan due to private sector and developmental project interventions. These two provinces can be explored for future hybrid maize production due to vast areas of virgin lands. As the price of maize hybrid seeds is high in Pakistan, smallholder farmers cannot afford to purchase it, which in turn force them to grow open-pollinated varieties.

About 80 percent of Pakistani farmers are smallholders, and their limited ability to purchase maize hybrid seeds is one of the reasons for the poor adoption of hybrid seeds. The provision of subsidies for hybrid maize seed, along with improvements in the delivery channels and extension services, may help smallholder farmers adopt maize hybrids. Hybrid maize variety dissemination should target non conventional areas (the local production of maize hybrid seeds will help to reduce retail prices) in Pakistan to scale the maize production at the local level. The Agricultural Innovation Program (AIP) has introduced a large number of maize hybrids through public and private sector seed companies to support Pakistan in scaling the adoption of hybrid maize seed and enhance yield.

A study in 2008 found that the inadequate application of inputs, as well as the poor selection of suitable varieties for given ecologies, contributed to lower yields [4]. By introducing maize hybrids, yield can be doubled within the same area. In Pakistan, the area under maize hybrid cultivation is comparatively low [5]. As changing climatic conditions are adversely affecting maize yields, it is crucial to focus on the promotion of climate resilient maize hybrids [6]. Through the adoption of these hybrids, the maize yield can be increased substantially [7–9].

In the past, a number of studies have found the adoption of hybrid maize have a positive impact on household welfare [5, 10, 11–17]. Using propensity score matching and endogenous switching regression, Khonje et al. [12], found that hybrid adopters reaped a higher yield, consumption, and food security. Hybrid adoption is viable and profitable in Nigeria [13]. Becerril and Abdulai [18] examined the adoption of the improved maize germplasm in Oaxaca and Chiapas in Mexico, and found that hybrid maize adoption had a positive impact on household welfare. In Malawi, a study found that hybrids were adopted by farmers interested in higher yields and drought-resistant attributes, while OPVs were mostly adopted by farmers interested in early maturity [11]. Drought-Tolerant Maize (DTM) hybrid is more profitable compared with open-pollinated varieties, and also offers resilience to changing climatic conditions [16]. DTM has the capacity to generate enormous amount of cumulative benefits to both producers and consumers in developing countries, thereby contributing to Sustainable Development Goals (SDGs). Although maize hybrids provide higher yield compared with OPVs, there are several barriers to the adoption of maize hybrids, such as high prices and nonavailability of the seed [14].

None of these existing studies, however, focused on the impact of hybrid maize seeds in Pakistan. Hence, this study is the first attempt to examine the factors influencing the adoption of hybrid maize and its impact on maize yields and household income levels in Pakistan. The rest of the paper is organized as follows: in Section 2, the conceptual framework is presented.  Section 3 presents methodology. In Section 4, the data and description of variables are presented, and Section 5 outlines the empirical results. The paper concludes with some policy recommendations in Section 6.

## 2. Conceptual Framework

**Figure 1:**
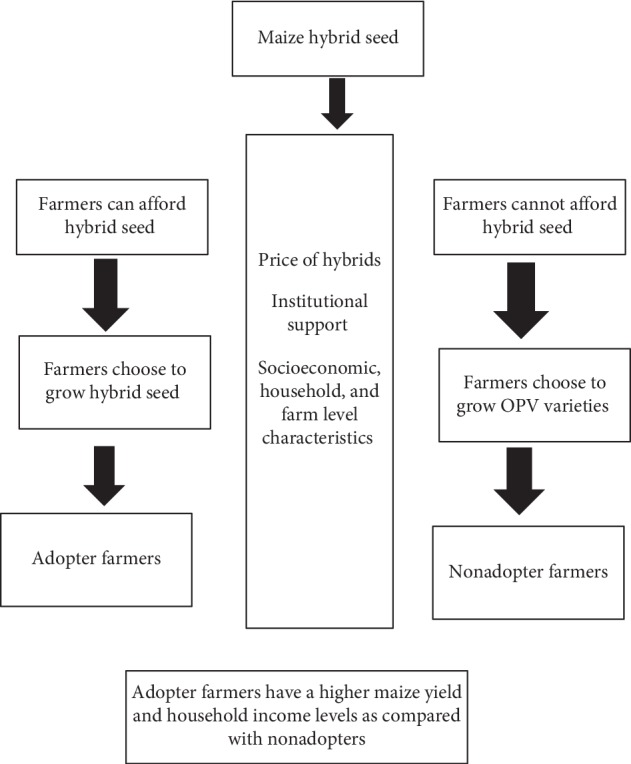
Conceptual Framework. We consider that maize farmers have two options i.e., 1 either to grow the maize hybrids or OPVs; However this choice is not as simple as many factor are actually influencing this choice of maize hybrids. The conceptual framework shows that price of the hybrid seed and lack of information from agricultural extension services are the major constraints in adoption of the maize hybrid seed. However the adopters have higher maize yield, higher income levels as well as higher overall welfare.

We consider that maize farmers have two options: grow either maize hybrids or OPVs. However, this choice is complex as several factors influence this decision. The conceptual framework presented in Figure 1 shows that besides wealth and education of the farmers, the price of hybrid seeds and lack of access to agricultural extension services are the major constraints in hybrid maize adoption.

## 3. Data, Sampling, and Methodology

### 3.1. Data and Sampling

This study aims to document the factors influencing the household decision to adopt hybrid maize and its impact on household welfare using primary data collected from farm households in all four major provinces of Pakistan: Punjab, Sindh, KPK, and Balochistan. Using detailed questionnaires, we collected a wide range of information covering socioeconomics characteristics, access to institutions, and infrastructure from both categories of farmers, i.e., adopters and nonadopters of hybrid maize. A multistage sampling procedure was adopted for the sampling of farm households for the study. In the first stage, we selected four provinces of Pakistan (Punjab, Sindh, KPK, and Balochistan). In the second stage, we selected three districts from each province, and in the third, we collected information from 822 farmers, including both adopters and nonadopters.

### 3.2. Econometric Methods

For the analysis of the factors influencing the adoption of hybrid maize varieties, we used the bivariate probit model as the dependent variable as primary, i.e., 1 if the farmer adopted the hybrid maize variety and zero otherwise. We used the propensity score matching (PSM) approach to analyze the impact of the adoption of hybrid maize on yield, income, and poverty level. The PSM creates the conditions of the randomized controlled experiment and then matches similar adopters with similar nonadopters (the propensity score matching (PSM) approach can be implemented by employing a number of matching algorithms, i.e., nearest neighbor matching (NNM), kernel-based matching (KBM), radius matching (RM), and Mahalanobis metric matching (MMM)), which helps correct sample selection bias.

## 4. Description of Variables

The description of the variable used in this paper along with the descriptive statistics is presented in Table 1. As in most developing countries, the farmers are middle aged, with the mean age being about 43 years. The average family size in the study area was quite high (around 11 members per family), and a large number of farmers were living in joint family systems (approximately 86%). In the joint family system, farmers carry out farming operations/practices jointly, which means that joint family systems tend to have adequate family labour for farming activities.

The mean education of the farmers was about 9.7 years of schooling, which is a reasonably good level of education. The average years of experience in agriculture was about 19 years, while the experience of growing maize was 17 years. Approximately 68% of the farmers owned land, and the average landholding was about 14 acres. About 32% of the farmers were tenant farmers, and they paid a rent of Pakistani Rupee (PKR) 32,935 per acre, which varied according to crop yield and climatic conditions. About 46% of the households owned a tractor, 33% owned a tubewell, and only 1% owned a zero tillage drill. Television, an important source of information, was owned by about 91% of the households.

Access to infrastructure and facilities plays an important role in technology adoption. Among the sampled farmers, we found that 88% had a metal road in the village, 36% had a basic health unit in the village, and only 7% had access to an agricultural extension office.

Table 2 shows the cost of production of OPV and hybrid maize. The cost of production of OPV was PKR 25,890, while the cost of production of the hybrid was PKR 34,981. The cost of hybrid maize is high mainly due to a higher cost of the seed; the other operational costs are almost the same for both OPV and hybrids. The cost-benefit ratio for OPV varieties was 2.10, while it was 2.51 for the hybrids. The per acre net profits from the OPV cultivation was PKR 28,562, while it was PKR 52,519 from the cultivation of the hybrids. Our findings on the cost-benefit analysis are similar to the previous studies by Kumar et al. [6].

## 5. Empirical Results

### 5.1. Factors Influencing the Adoption of Hybrid Maize

As the dependent variable is bivariate discrete (i.e., 1 if the households adopted the maize hybrids; 0 otherwise), we estimated a probit model to identify factors driving the adoption of the hybrid maize and presented the results in Table 3.

Farmers' status was included as a dummy variable, and the coefficient is positive and highly significant at a 1 percent level of significance, indicating that farmers who own land are more likely to adopt maize hybrids as compared with tenants. Land owner farmers have geater probability of adopting the maize hybrids than tenant farmers because tenant farmers do not have financial capacity to afford the high price of the maize hybrid seeds. In addition, tenants have to share their output with the landlord, and net benefits are lower for investments for tenants; hence, they have less incentive to invest on inputs including the hybrid seed. The age coefficient is negative and significant at a 5 percent level of significance, which implies that young farmers are more likely to adopt maize hybrids as compared to older farmers. This could be because young farmers have more awareness and are more likely to try to new technology compared with older farmers. Years of schooling was positive and highly significant at a 1 percent level of significance, which means that farmers with a better level of education had a greater probability of adopting maize hybrids as compared with those with a lower level of education; hence, we can conclude that human capital plays a significant role in technology adoption and has a positive influence in scaling agricultural advancements in developing countries. The joint family system dummy (1 if the farmer lives in joint family; 0 otherwise) is positive and highly significant at a 10 percent level of significance, implying that farmers living in the joint family system 11 have a greater tendency to adopt maize hybrids compared with farmers living in a nuclear family system.

The size of the land owned by the farm household is positive and highly significant at a 1 percent level of significance, signifying that the farmers with larger landholdings were most likely to adopt maize hybrids, mostly for two reasons: (1) farmers with large landholdings were rich and able to invest in maize hybrid seeds which are expensive and (2) farmers with large landholding were able to maximize the output and revenue by investing in the new technology. We also included a few variables such as presence of metal road and access to extension services to investigate the role of infrastructure and institutions on technology adoption. The coefficient of the agricultural extension services was positive and significant at 5 percent level of significance, meaning that farmers with more contact with the extension department were more likely to adopt the hybrid (actually, the farmers who have contact with the agricultural extension department know about the benefits of maize hybrids). Household and agricultural assets such as ownership of a tractor or car were positive and significant, signifying the positive role of wealth and assets on technology adoption. The LR Chi-squared test was also positive and highly significant, indicating the robustness of the variables included in the model.

The use of simple probit analysis to investigate whether farmers adopt maize hybrids or not does not provide a clear picture of the scale of adoption. Therefore, we further estimated the farmsize under maize hybrids using the censored least absolute deviation (CLAD13) model, and the results are presented in Table 4. The results of area under maize hybrids cultivation are very much similar with the adoption results presented in Table 3. The human and physical capital of farm households positively influences the farmsize under maize hybrid cultivation. Access to infrastructure and institutions also drives maize hybrid technology adoption.

### 5.2. Factors Influencing Willingness to Pay for Hybrid Maize

To analyze the farmers' willingness to pay for hybrid maize seeds, we estimated the ordered probit model (which can be employed when there are various categories) and summarized the results in Table 5. The dependent variable for the estimation of the ordered probit were the available ranges: farmers willing to pay PKR 2000–3000 were ranked 1, farmers willing to pay in the range of PKR 3000–4000 were placed in rank 2, farmers willing to pay PKR 4000–5000 were placed in rank 3, and farmers willing to pay more than PKR 5000 were placed in the highest rank, rank 4.

The results show that the farmers with higher human and physical capital were willing to pay more for maize hybrid seeds. We found that land-owning farmers were willing to pay more for the hybrid maize seed as compared with tenant farmers because the entire benefit of adoption of hybrid maize seeds will accrue to the owner, while in the case of the tenant, only partial benefits will come to them, with some of the benefits flowing to the owner of the land. Level of education also positively drives farmers' willingness to pay for the maize hybrid technology. Access to agricultural extension services and asset ownership also play an important role in farmers' willingness to pay for maize hybrids.

From the above discussion, it is very clear that households with large landholdings, more assets and more human capital are most likely to adopt maize hybrids in Pakistan. Therefore, the price of hybrid seed needs to be reduced for small farmers with fewer resources and fewer assets.

### 5.3. Impact of Hybrid Maize Adoption on Household Welfare in Pakistan

The impact of hybrid maize adoption on household welfare was estimated by employing the propensity score matching approach (PSM), and the results are presented in Table 6. The PSM analysis was carried out by employing four different matching algorithms, i.e., nearest neighbor matching (NNM), kernel-based matching (KBM), radius matching (RM), and Mahalanobis metric matching (MMM) (it is always better to employ more than one matching algorithm as the robustness of the results across various algorithms can be checked). By employing PSM, the impact of maize hybrid seed adoption (average treatment affect of the treated-ATT) (ATT indicates the difference in outcome of the similar households having adopted maize hybrids with the similar households having nonadopted maize hybrids) was estimated on maize yield, household income, and poverty levels. The impact on maize yield was positive and highly significant, demonstrating that adopters have higher yields as compared to non-adopters. Similarly the impact on household income was also positive and highly significant, signifying that adopters had higher income levels. The impact on poverty levels was negative and significant which means that the adopters were likely to be living in less poverty. We also performed number of balancing tests to check the matching quality (although the matching quality was checked, the results are not reported here; the tests employed to check the matching quality include median absolute bias before and after matching, the joint significance of covariates before and after matching, and the value of R-square before and after matching). The results are inline with the previous studies (for example, [19]).

Despite estimating the impact of the maize hybrids on the yield and poverty, it is also crucial to estimate the efficiency levels of the hybrid versus OPVs. We used the Frontier 4.1 software to estimate the technical, allocative, and economic efficiency (the economic efficiency is actually a product of the technical efficiency and allocative efficiency) of the maize producers and presented the result in Table 7. The mean technical efficiency of the hybrid producers was 0.87, while the mean technical efficiency of the OPV producers was 0.75. The mean allocative efficiency of the hybrid producers was 0.84, while the mean allocative efficiency of the OPV producers was 0.71. The mean economic efficiency of the hybrid producers was 0.73, while the mean economic efficiency of the OPV producers was 0.53. The difference in technical, allocative, and economic efficiency levels of the hybrid and OPV producers indicates that there are some differences in the efficiency levels of the hybrid and OPV producers.

From the empirical findings, it can be concluded that adoption of the maize hybrids has a positive and significant impact on maize yield, household income, and overall household welfare. The technical, allocative, and economic efficiency levels of the hybrid maize adopters are also high compared with nonadopters. The affordability and accessibility of the maize hybrids needs to be ensured for small farmers. Hybrid maize seed prices need to be reduced through local production, as currently they are mostly imported.

## 6. Conclusion and Policy Recommendations

This study is among the few focusing on hybrid maize adoption in Pakistan. The empirical results show that the adoption of maize hybrids leads to increases in yield and income and lowering of poverty levels, thereby resulting in an improvement in the wellbeing of the farmers. However, there are several constraints to adoption, particularly affordability and lack of knowledge. Human capital such as education, institutional support, agricultural extension services, as well as household assets drives the adoption of hybrid maize. Educated and wealthy farmers are more likely to adopt the new technology; hence, there is a need to invest in human capital development either through formal education or training on recent development of new agricultural technology. The farmers' willingness to pay for the maize hybrids also indicated that rich farmers were willing to pay more for the hybrid seed, while the resource poor farmers were less likely to pay for the maize hybrid seed. Hence, to increase the adoption rate of the maize hybrids, either the ability of the poor farmer to pay for the seeds should be enhanced or the seed price be lowered.

Pakistan imports over 50% of the hybrid seeds, which translates to a higher retail price. Hence, building the competitiveness of local seed companies, incentives and tax Grace for companies involved in local hybrid seed production, fast tracking the release process of new varieties, and providing technical support for hybrid seed production are among the major areas that need policy interventions.

## Figures and Tables

**Table 1:** Data and description of variables.

Characteristics	Description	Mean	Std. dev.
*Demographic and household*			
Age of farmer	Age of the farmer (in number of years)	42.53^*∗∗*^	10.54
Family size	Total number of family members	11.09	4.25
Family system	1 if the households are living in a joint family	0.86^*∗*^	0.39
Marital status	1 if the respondent is married, 0 otherwise	0.56	0.44

*Education and experience*			
Education of farmer	Schooling of the farmer (in years)	9.70^*∗∗*^	4.42
Farming experience	Farming experience (in number of years)	19.10^*∗*^	10.21
Experience in growing maize	Experience in growing maize (in number of years)	16.64^*∗*^	9.24

*Farm and household assets*			
Tenancy (farmer status)	1 if farmer is owner, 0 for tenant	0.68	0.35
Land owned	Land owned (in number of acres)	14.23^*∗∗*^	30.54
Land rental (PKR/acre)	Average land rental charges (in pakistani rupees per acre per year)	32,935	8080
Tractor	1 if household owns a tractor, 0 otherwise	0.46^*∗∗*^	0.51
Tubewell	1 if the tubewell is owned by the household, 0 otherwise	0.33^*∗*^	0.38
Television	1 if the household has television, 0 otherwise	0.91^*∗*^	0.28
Zt drill	1 if household own zero tillage drill, 0 otherwise	0.01^*∗*^	0.11
Car	1 if household own car, 0 otherwise	0.20	0.16

*Access to infrastructure and institutions*			
Metal road	1 if there is a metal road in the village, 0 otherwise	0.88	0.32
Basic health unit	1 if there is a basic health unit in the village, 0 otherwise	0.36	0.39
Agri. Extension	1 if the farmer receives agricultural extension services, 0 otherwise	0.07	0.12

*Province (location)*			
Punjab	1 if the respondent is from Punjab, 0 otherwise	0.34	0.27
Sindh	1 if the respondent is from sindh, 0 otherwise	0.33	0.26
KPK	1 if the respondent is from KP, 0 otherwise	0.33	0.26
Balochistan	1 if the respondent is from balochistan, 0 otherwise	0.10	0.09
Household income	Income of the household in pakistani rupees	24850	1236
OPV yield	OPV yield in tons per hectares	5.7	4.2
Hybrid yield	Hybrid yield in tons per hectares	7.6	6.8
Poverty	Headcount index of poverty	0.08	0.05

*Note.* The results ^*∗∗∗*^, ^*∗∗*^, ^*∗*^ indicates that there are significant differences between the adopters and nonadopters.

**Table 2:** Comparison of the cost of production (hybrid vs OPV).

Operation	OPV	Hybrid	Difference	*t*-values
Cost of production (PKR)	25,890	34,981	–9091^*∗∗*^	2.34
Average yield (ton)	5.7	7.6	–1.9^*∗*^	1.72
Revenue per acre	54,452	87,500	–33,048^*∗∗∗*^	2.55
Net profit	28,562	52,519	–23,957^*∗∗∗*^	2.86
Cost-benefit ratio	2.10	2.51	–0.41	1.23

*Note.* Results are significant at ^*∗∗∗*^, ^*∗∗*^, ^*∗*^ 1, 5 and 10 percent levels respectively. OPV stands for the open-pollinated varieties.

**Table 3:** Determinants of the hybrid adoption (probit estimates).

Variable	Coefficient	*t*-values
*Demographic and household*		
Age of the farmer	–0.01^*∗∗*^	2.23
Family size	0.01^*∗*^	1.70
Family system (dummy)	0.02^*∗*^	1.83

*Human capital*		
Education	0.01^*∗∗∗*^	2.45

*Farm and household assets*		
Farmer status (dummy)	0.02^*∗∗∗*^	2.56
Land owned (in acres)	0.02^*∗∗∗*^	2.86
Own tractor (dummy)	0.01^*∗∗∗*^	2.57
Own car (dummy)	0.02	1.44
Own televion (dummy)	–0.02	–1.38

*Access to infrastructure/institutions*		
Metal road (dummy)	–0.03	–1.55
Agriculture extension (dummy)	0.02^*∗∗*^	2.04

*Location (province)*		
Punjab (dummy)	0.05^*∗∗∗*^	2.71
Sindh (dummy)	–0.02	–1.45
KPK (dummy)	0.03^*∗∗*^	2.01
Constant	0.031	1.42
*R*-square	0.37	
LR-chi square	128.34	
Prob > chi square	0.000	
Total number of observations	822	

*Note. Results* are significant at ^*∗∗∗*^, ^*∗∗*^, ^*∗*^ 1, 5 and 10 percent levels respectively.

**Table 4:** Number of hectares under hybrid (CLAD estimates).

Variables	Coefficient	*t*-values
*Demographic and household*		
Age of the farmer	0.02^*∗*^	1.84
Family size	0.03^*∗*^	1.71
Joint family system (dummy)	0.01	1.43
*Human capital*		
Education	0.01^*∗∗*^	2.36

*Farm and household asset*		
Tenancy (farmer status) (dummy)	–0.01	–1.32
Land owned (acres)	0.11^*∗∗∗*^	2.49
Own tractor (dummy)	0.04^*∗∗∗*^	2.34
Own tubewell (dummy)	–0.17	–1.23
Own zt drill (dummy)	0.03^*∗*^	1.91
Own car (dummy)	0.01^*∗∗*^	2.16
Own television (dummy)	0.01^*∗∗*^	2.04

*Access to infrastructure and institutions*		
Access to basic health unit (dummy)	–0.03	–1.26
Access to agricultural extension (dummy)	0.02^*∗∗∗*^	2.65

*Location (province)*		
Punjab (dummy)	0.03^*∗*^	1.78
Sindh (dummy)	0.01	1.27
KPK (dummy)	0.02^*∗∗∗*^	2.41
Constant	0.03	2.12
*R*-square	0.53	
LR-chi square	286.34	
Prob > chi square	0.000	
Total number of observations	822	

*Note. Results* are significant at ^*∗∗∗*^, ^*∗∗*^, ^*∗*^ 1, 5 and 10 percent levels respectively. CLAD stands for the censored least absolute deviation model.

**Table 5:** Farmers' willingness to pay (WTP) for hybrid maize seed (ordered probit estimates).

	Rank 1 (PKR 2000–3000)		Rank 2 (PKR 3000–4000)		Rank 3 (PKR 4000–5000)		Rank 4 (PKR >5000)	
Variable	Coef	*t*-value	Coef	*t*-value	Coef	*t*-value	Coef	*t*-value
Farmer status	0.01^*∗∗∗*^	2.89	0.02^*∗∗*^	2.13	0.04^*∗∗*^	2.16	0.06^*∗*^	2.82
Age of the farmer	0.02^*∗*^	1.83	–0.01^*∗∗*^	–2.19	–0.03	–2.00	–0.05^*∗∗*^	–2.35
Education	0.02^*∗∗∗*^	2.90	0.03^*∗*^	1.80	0.05^*∗*^	1.92	0.03^*∗∗*^	2.13
Family system	0.03^*∗∗*^	2.11	0.03	1.61	0.01	1.35	0.02^*∗∗*^	2.08
Marital status	–0.04	–0.80	–0.02	–1.25	–0.02	–1.39	0.03	1.44
Own land	0.01^*∗*^	1.75	0.02^*∗*^	1.66	0.05^*∗*^	1.91	0.04^*∗*^	1.67
Family size	0.07	1.44	0.04	1.39	0.02	1.42	0.06	1.50
Metal road	0.03	1.20	0.02	1.35	0.02	1.46	0.02	1.25
Agri. extension	0.04	1.40	0.03	1.53	0.04	1.27	0.04	1.63
Tractor ownership	0.01^*∗∗*^	2.36	0.05^*∗∗∗*^	2.77	0.01^*∗∗*^	1.99	0.01^*∗∗∗*^	2.73
Car ownership	0.05	1.52	0.03^*∗*^	1.91	0.03^*∗*^	1.74	0.04^*∗∗*^	2.11
Televisin ownership	0.03	1.48	0.02^*∗*^	1.75	0.04^*∗*^	1.66	0.03	1.53
Punjab	0.04^*∗∗∗*^	2.87	0.03^*∗∗∗*^	2.53	0.02	1.55	0.03^*∗*^	1.58
Sindh	0.01	1.56	0.02	1.47	0.03	1.59	0.01	1.27
KP	0.03^*∗*^	1.72	0.03	1.62	0.01	1.42	0.03	1.28
Constant	0.03^*∗*^	1.68	0.02	1.44	0.02	1.53	0.03	1.36
*R*-square	26.31							
LR–Chi square	155.64							
Prob > chi square	0.000							
Total number of observations	822							

*Note. Results* are significant at ^*∗∗∗*^, ^*∗∗*^, ^*∗*^ 1, 5 and 10 percent levels of significance.

**Table 6:** Impact of hybrid adoption on maize yield, household income and poverty levels.

Matching algorithms	Outcome	Caliper	ATT	*t*-values	Critical level of hidden bias	Nos of treated	Nos of control
NNM	Maize yield	0.01	2.41^*∗∗*^	2.37	1.25–1.30	234	467
	Household income	0.03	2176^*∗*^	1.92	1.50–1.55	234	467
	Poverty	0.08	–0.03^*∗∗*^	2.14	1.05–1.10	234	467

KBM	Maize yield	0.05	2.35^*∗∗∗*^	2.50	1.30–1.35	316	425
	Household income	0.001	3122^*∗∗*^	2.13	1.65–1.70	316	425
	Poverty	0.003	–0.02^*∗*^	1.87	1.25–1.30	316	425

RM	Maize yield	0.002	3.11^*∗∗*^	2.62	1.15–1.20	289	403
	Household income	0.003	3518^*∗∗∗*^	2.66	1.20–1.25	289	403
	Poverty	0.002	–0.03	1.22	–	289	403

MMM	Maize yield	0.004	2.62^*∗∗∗*^	3.05	1.35–1.40	240	367
	Household income	0.002	2936^*∗∗*^	2.04	1.25–1.30	240	367
	Poverty	0.07	–0.03^*∗∗*^	2.15	1.25–1.30	240	367

*Note.* NNM stands for the nearest neighbor matching, KBM stands for the kernel based *matching*, RM stands for the radius matching, MMM stands for the Mahalanobis metric matching. ATT stands for the average treatment affect for the treated. The results are significant at ^*∗∗∗*^, ^*∗∗*^, ^*∗*^ 1, 5 and 10 percent levels respectively.

**Table 7:** Technical, allocative and economic efficiency of maize producers (hybrid vs OPV).

Efficiency	Hybrid			OPV		
	Mean	Minimum	Maximum	Mean	Minimum	Maximum
Technical	0.87	0.76	0.93	0.75	0.67	0.84
Allocative	0.84	0.73	0.90	0.71	0.62	0.79
Economic	0.73	0.55	0.84	0.53	0.41	0.66

*Note.* Mean *levels* of the technical, allocative and economic efficiency has been reported.

## Data Availability

The data used to support the findings of this study are available from the corresponding author upon request.
